# Economics of compliance for mixing allergy immunotherapy

**DOI:** 10.3389/falgy.2025.1729120

**Published:** 2026-01-13

**Authors:** Katarina I. Trapanotto, Mary F. Lee-Wong, Robert A. Promisloff, Anthony M. Szema

**Affiliations:** 1 Stony Brook University School of Nursing, Stony Brook, NY, United States; 2 Icahn School of Medicine at Mount Sinai, New York, NY, United States; 3 Maimonides Medical Center, Brooklyn, NY, United States; 4 Drexel University College of Medicine, Philadelphia, PA, United States; 5 Donald and Barbara Zucker School of Medicine at Hofstra/Northwell, Hofstra, NY, United States

**Keywords:** Allergenic Extracts Compounding Area (AECA), Primary Engineering Control (PEC) Hood, United States Pharmacopeia (USP), chapter 797, allergenic extract, immunotherapy, economics, costs analysis

## Abstract

United States Pharmacopeia (USP), a nonprofit organization that sets safety standards for food, medicine and supplements updated its Chapter 797 standards for the preparation of allergy immunotherapy vials in November 2023. These guidelines impacted facility and personnel qualifications, workflow, and laboratory documentation. We hypothesized adhering to regulations may be financially difficult for allergy practices. To comply, offices must either use mail order prescription facilities or allocate financial resources to purchase a Primary Engineering Control (PEC) Hood, a device used to protect against airborne contaminants, or build-out an Allergenic Extract Compounding Area (AECA), a room specialized for compounding allergens. We performed a comparative analysis to determine financial feasibility of these three scenarios with the support of a Public Service Enterprise Group (PSEG) grant. Of these three options prescription sets were most costly, followed by PEC Hood and AECA. For both PEC Hood and AECA, costs were recouped by the third week of operation. In conclusion, for long term operation, the AECA would be most feasible for small private practice outpatient clinics in regard to overall cost and estimated revenue.

## Introduction

USP is an independent scientific nonprofit organization that addresses quality assurance, enhancing regulatory predictability and is involved with manufacturers' medicine distribution (1). USP regulates dietary supplements, medications, and healthcare quality with routine updating.

Chapter 797 regulates the sterility of labeled compounded preparations since failure to adhere could induce harm or death (2). Compliance entails compounding in an AECA or inside a PEC. AECA regulations require a visible perimeter with sufficient lighting, temperature and humidity controls, no carpeting or dust collecting overhangs. All surfaces, fixtures, and shelving must be free of crevices and are mandated to be cleaned by 70% sterile isopropyl alcohol. While compounding in the AECA room, only authorized personnel donning PPE (Protective Personal Equipment) can be present (3). ISO (International Organization of Standards) Class V PEC requires biannual certification and disinfection with 70% sterile IPA. Both AECA and PEC must be located away from restrooms, warehouses, or food prep areas and cannot be adjacent to unsealed windows, doors that connect outdoors, nor near busy staff traffic. AECA and PEC must be at least one meter away from sinks.

## Methods

A comparative analysis was conducted to determine which option was most economically viable for an allergy practice: (1) ordering prescription allergen extract sets from vendors (2) installation of PEC, (3) construction of AECA. Prescribed allergen extracts costs from Stallergenes Greer™ were used for calculations. PEC estimate included fume hood and certification cost. AECA cost entails room conversion and materials.

Prescription allergen extract costs, derived from Stallergenes Greer™, included a five-vial starter set containing 5 mL vial of maintenance concentrate plus four-step down dilution vials plus shipping. New York state insurance reimbursement was included to calculate final net cost.

PEC cost included an 18-inch PEC Hood model from Sentry Air Systems plus certification and inspection quotes from Technical Safety Services Inc.

Materials for the AECA included, bacteriostatic curtains with track, class 100 pre-saturated wipes with sterile 70% IPA (isopropyl alcohol), cleanroom bouffant, washable ceiling tiles, humidifier/dehumidifier, temperature and humidity monitor, industrial nitrile gloves, MERV-13 (Minimum Efficiency Reporting Value) HEPA (High-Efficiency Particulate Air) filter, standard shoe covers, surgical masks, isolation gowns, laminate countertops with storage, licensed contractor, Ultraviolet-C plug-in air sanitizer, pre-catalyzed waterborne epoxy paint, and sheet rock (Table 1).

**Table 1:** Materials and construction costs for AECA.

AECA Materials and Construction Cost		
Bacteriostatic Curtains with Track	2 Sets	$700.00
Class 100 Pre-Saturated Wipes Sterile 70% IPA	24 Packs, 30 wipes per pack	$192.00
Cleanroom Bouffant	5–8 Cases, 1,000 per case	$350.00
Gypsum Ceiling Tiles	3 Cases, 48 tiles	$190.00
Humidifier/Dehumidifier	1 Unit	$90.00
Humidity and Temperature Monitor	1 Unit	$45.00
Industrial Nitrile Gloves	1 Case, 1,000 pairs	$45.00
MERV-13 HEPA Filter	1 Unit	$65.00
Sealed Alarm Window	Preexisting	$0.00
Standard Shoe Covers	2 Cases, 300 per case	$70.00
Surgical Masks	2 Boxes, 100 per box	$40.00
Isolation Gowns	3–4 Cases, 50 per case	$265.00
Laminate Countertops with Storage	Quote per Contractor	$10,000.00
Licensed Contractor	1	$4,000.00
UV-C Plug-in air Sanitizer	I Unit	$64.00
Pre-Catalyzed Waterborne Epoxy Paint	2 Gallons	$103.00
Drywall	Quote per Contractor	$2,500.00
Estimated Total Cost		$18,719.00

## Results

**Figure 1:**
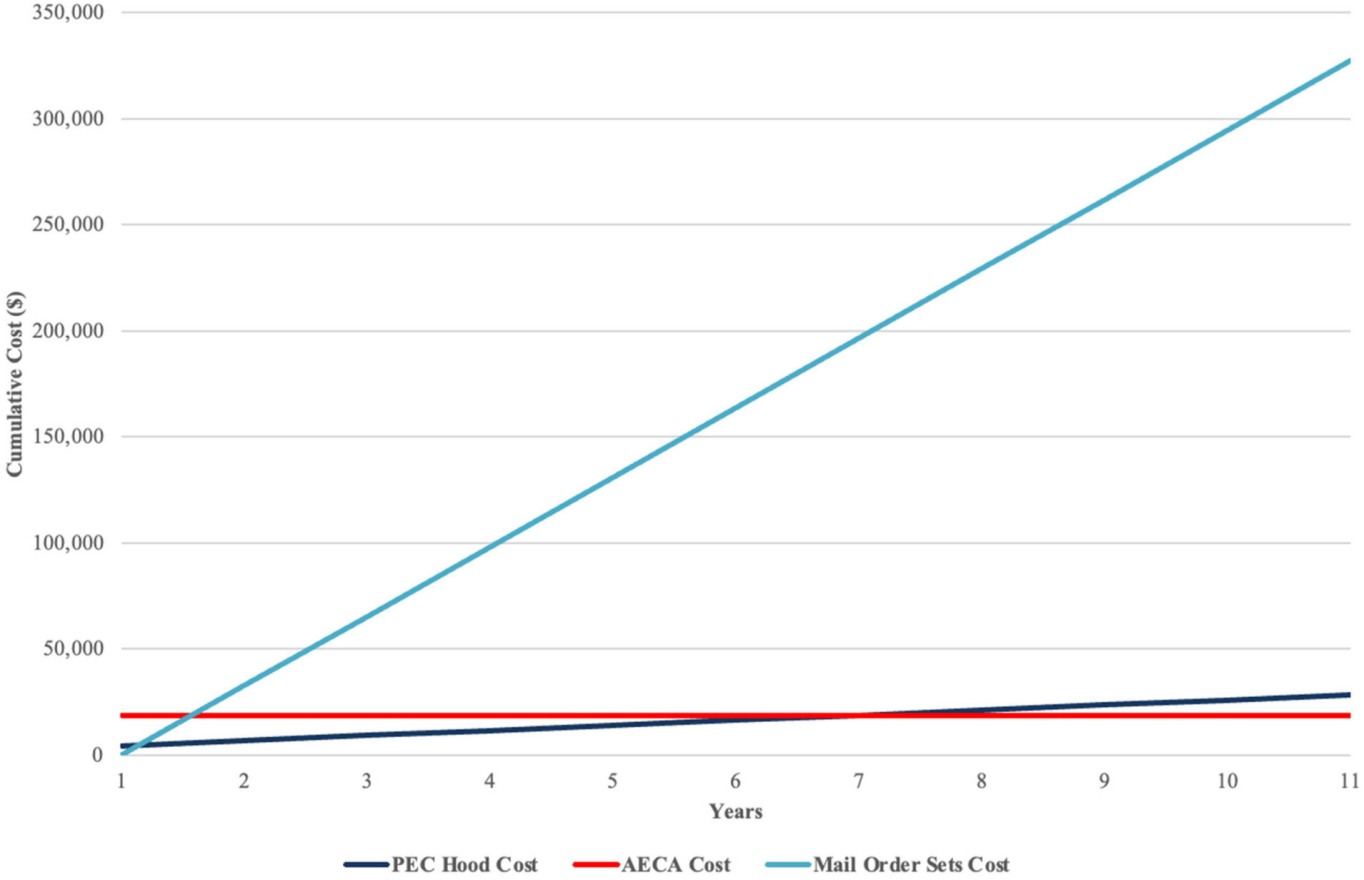
Graph comparing the long term compliance of AECA and PEC hood, demonstrating PEC hood exceeding AECA costs by year seven.

Of the three allergenic manufacturers in the United States, Stallergenes Greer™, HollisterStier Allergy™, and ALK Allergy™, only Stallergenes Greer™ ships patient-prescription bottles to medical offices. A five-vial starter set consisting of one 5 mL maintenance bottle plus respective dilutions will vary in cost depending on the number of antigens included eg: less than 6 antigens is priced at $498.53, 7–12 antigens $565.43, and 13–15 antigens $660.15 (4). Pricings do not include shipping cost, which is an additional estimated $80–100 for priority overnight shipping (4). Total equates to $598.53, plus shipping. Prices are effective until March 1st, 2025. New York median reimbursement rate for three allergens from two allergy practices is approximately $300. Result is a net loss of $298.53 for every three allergen sets ($598.53–$300=$298.53), indicating that ordering prescription allergy sets is not economically feasible (Figure 1).

PEC incurred a total cost of $4,376.00 ($1,976.00, hood cost, +$2,400, annual certification cost). Compliance requires particle count, air flow visualization as well as both surface and airborne colony sampling biannually.

AECA's cost was approximately $18,719.00. Costs did not include the allergen extracts, wages nor personnel training. AECA rooms need to maintain sterility, but no certification required.

## Discussion

Long term financial feasibility needs to be cognizant of income vs. expenses to determine whether an allergy practice survives. Allergy offices treat a median of fifty immunotherapy patients a week (5). Registered nurses hourly wage of $45.42 for a 30-h week is a total $1,362.60 per week (6, 7). Stallergenes Greer™ 5 mL allergenic vial costs within a range of $110–$340, yielding a cost of $5,500–$17,000 for fifty patients, thus a 20-week net loss of $32,740.

**Figure 2:**
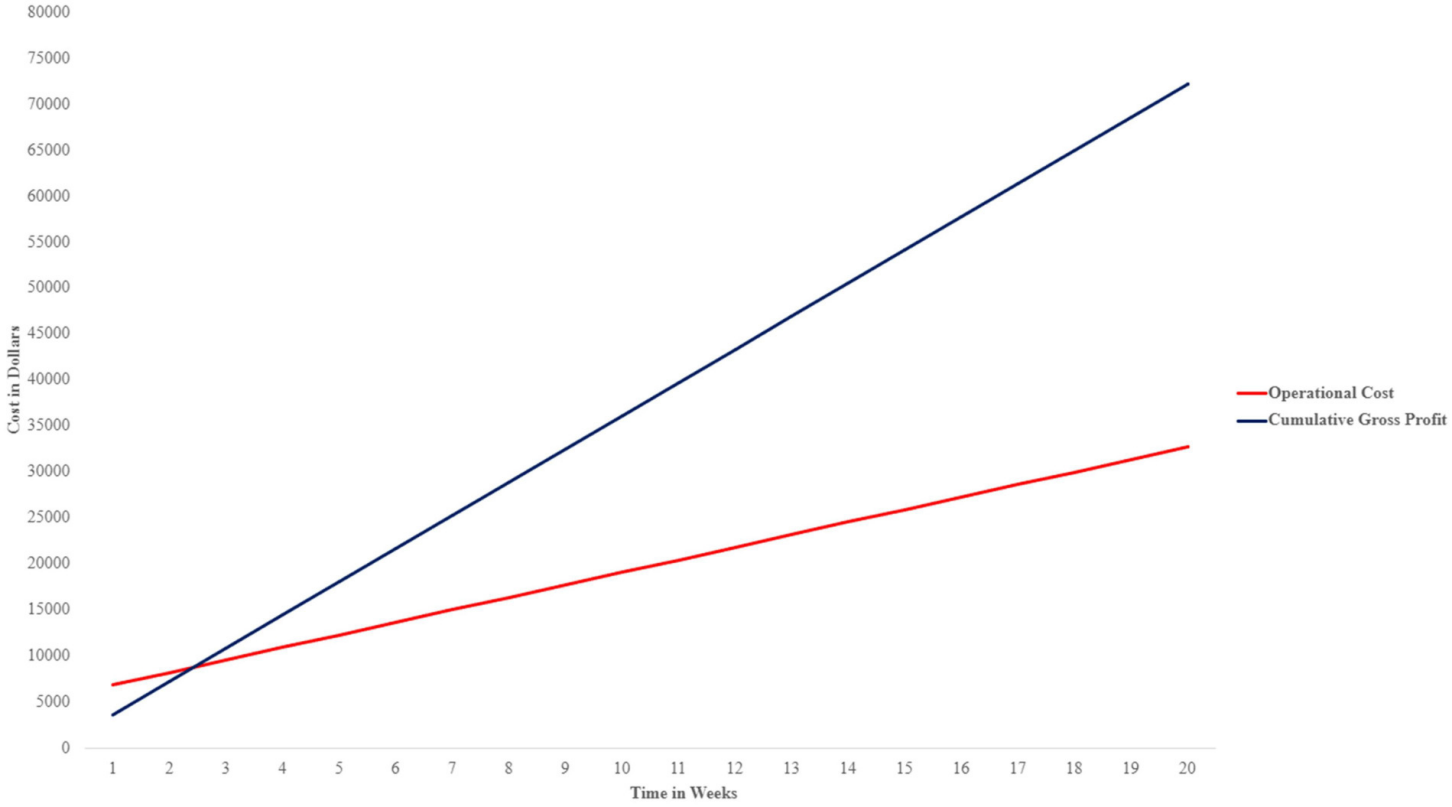
Graph illustrating financial viability of immunotherapy services in outpatient allergy clinics, showcasing net profits over operational costs.

CPT (Current Procedural Terminology) codes used for allergy immunotherapy reimbursement include: 95,115-one-injection, 95,117-multiple-injections, 95,165-vial-preparation-non-venom-antigens, and 95,144-single-dose-vial-preparation (8). Reimbursement median data from a New York City Community Hospital for one visit was $24.56 (Table 2). Self-pay patients spend a range of $120–$200 per visit depending on the number of allergy shots. This cost covers the vial and the administration of the shot. In addition, the $24.56 worth of billing will be reimbursed for every visit. For a 20-week period for fifty patients, using the lower end of the range of $120 and reimbursement, the gross profit yields $72,280. Using the initial cost and the gross profit, the total profit is $39,540 (Figure 2). Total profits do not account for other forms of procedures that incur revenue, and total costs do not account for cleaning materials, office space costs, electricity and office maintenance.

**Table 2:** Reimbursement rates for common CPT codes.

CPT Code	Description	2023 RVU	2023 National Payment Amount	Percent Change 2023–2024	2024 RVU	2024 National Payment Amount
95115	Immunotherapy, one injection	0.3	$10.17	−0.15%	0.31	$10.15
95117	Immunotherapy, two or more injections	0.35	$11.86	2.15.%	0.37	$12.12
95144	Antigen therapy services (single dose vial)	0.5	$16.94	−3.37%	0.5	$16.37
95165	Professional services for the supervision of preparation and provision of antigens for allergen immunotherapy; single or multiple antigens	0.45	$15.25	−5.52%	0.44	$14.41

Data is sourced from CMS medicare reimbursement rates.

**Figure 3:**
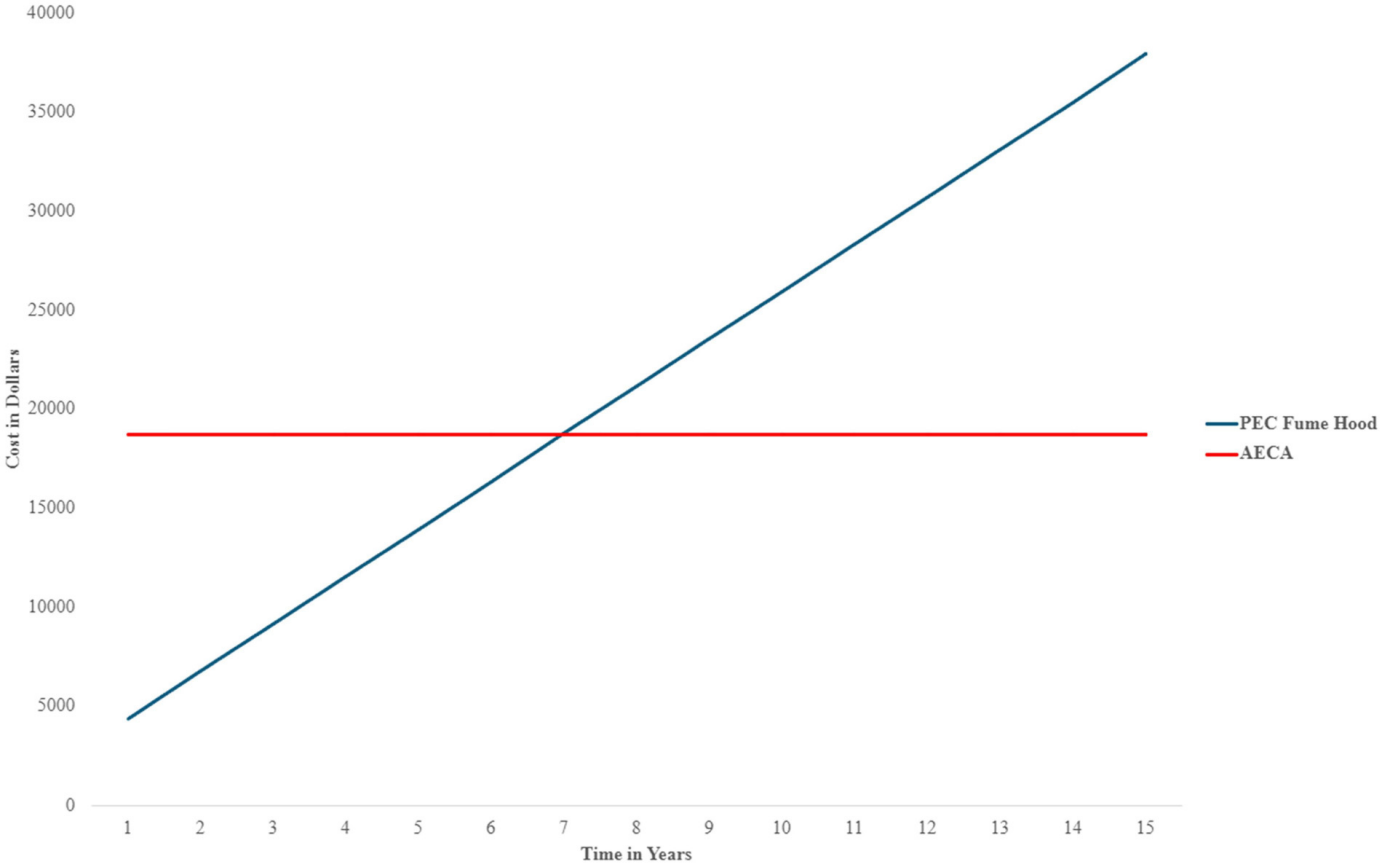
Demonstrates the cost for the PEC hood, AECA, and mail order sets, showing mail order sets the costliest.

Mail-order prescriptions set incurred a net loss of $200 per set. One-year PEC cost was $4,376.00 vs. $18,719.00 costs for AECA. Initially, PEC was less costly than AECA but, year seven, PEC cost exceeded AECA due to maintenance and certification expenses. AECA and PEC costs are recouped by immunotherapy profits (Figure 3). Continued AECA operation requires additional cleaning materials and PPE. Prices are subject to change.

After a 3-week period of operation, calculated revenue exceeded both AECA and PEC cost, thus recuperating initial investments (Figure 2).

## Data Availability

The original contributions presented in the study are included in the article/Supplementary Material, further inquiries can be directed to the corresponding author.
